# Comparing marine distribution maps for seabirds during the breeding season derived from different survey and analysis methods

**DOI:** 10.1371/journal.pone.0201797

**Published:** 2018-08-29

**Authors:** Alex Sansom, Linda J. Wilson, Richard W. G. Caldow, Mark Bolton

**Affiliations:** 1 Centre for Conservation Science, Royal Society for the Protection of Birds, Scottish Headquarters, Edinburgh, United Kingdom; 2 Centre for Conservation Science, Royal Society for the Protection of Birds, Inverness, United Kingdom; 3 Natural England, Blandford Forum, Dorset, United Kingdom; 4 Centre for Conservation Science, Royal Society for the Protection of Birds, Sandy, United Kingdom; CEFE, FRANCE

## Abstract

Understanding how seabirds use the marine environment is key for marine spatial planning, and maps of their marine distributions derived from transect-based surveys and from tracking of individual bird’s movements are increasingly available for the same geographic areas. Although the value of integrating these different datasets is well recognised, few studies have undertaken quantitative comparisons of the resulting distributions. Here we take advantage of four existing distribution maps and conduct a quantitative comparison for four seabird species (black-legged kittiwake *Rissa tridactyla*; European shag *Phalacrocorax aristotelis*; common guillemot *Uria aalge*; and razorbill *Alca torda*). We quantify the amount of overlap and agreement in the location of high use areas identified from either tracking or transect samples and use Bhattacharyya’s Affinity to quantify levels of similarity in the general distribution patterns. Despite multiple differences in the properties of the datasets, there was a far greater degree of overlap than would be expected by chance, except when adopting the most constrained definition of high use. Distance to the nearest conspecific colony appeared to be an important driver of the degree of similarity. Agreed areas of highest use tended to occur close to colonies and, with increasing distance from colonies, similarity between datasets declined and/or there was similarity in respect of their being relatively low usage. Interpreting reasons for agreement between data sources in some areas and not others was limited by an inability to control for the multiple potential sources of differences from both the sampling and modelling processes of the underlying datasets. Nevertheless, our quantitative comparative approach provides a valuable tool to quantify the degree to which an area’s importance is corroborated across multiple datasets, and therefore confidence that an important area has been correctly identified. This can help prioritise where the implementation of conservation measures should be targeted and identify where greatest scrutiny is required of the potential adverse environmental effects of any planned anthropogenic activities.

## Introduction

Seabird populations are undergoing declines at a faster rate than any other avian group [1] and face multiple pressures at sea, including many of anthropogenic origin. Marine spatial planning should focus conservation efforts in the places of greatest ecological importance to these birds and identify the sea areas in which they are most vulnerable to various anthropogenic activities in order to minimise, avoid or mitigate possible impacts on them. Such planning requires a good understanding of birds’ use of the marine environment, and knowledge of the exposure of the different components of the population to pressures in different places. Unfortunately, spatial distributions of species in the marine environment are particularly challenging to determine by survey. However, the range and sophistication of techniques available to gather spatial distributional information is constantly changing as technology advances. The at-sea distribution of marine birds has traditionally been sampled using boat-based and aerial transect visual sampling surveys, using techniques largely developed since the early 1980s [2–4]. Into the new millennium, these have been complemented with increasing use of digital aerial sampling (video or photographic stills) which takes advantage of advances in high resolution photographic equipment [5–7]. Moreover, increasing miniaturisation and technological improvement of bird-borne devices such as GPS loggers has proved to be transformational in the amount of information available on the at-sea movements of individual birds [8, 9].

Analytical approaches and software to map marine bird distribution data have also become more advanced and more accessible. A range of species distribution modelling techniques are now available for spatially referenced abundance and occurrence data [10, 11] and techniques have been developed to deal with the large quantities of movement data now resulting from tracking studies and the analytical challenges they present [9, 12–15]. These techniques mean that population-level distributions can now be estimated based on the tracked movements of individuals e.g. [13, 16]. This leads to the relatively new situation where population-level seabird distributional information derived from the two contrasting sampling techniques of transects and tracking has become available for the same geographic area.

The properties of the data collected under different sampling methods will affect the interpretation of any derived spatial distribution maps. Transect data capture concurrent location information across all individuals observed at a fixed point in space [17, 18]. However, the individuals observed are of unknown provenance and usually of unknown breeding status. Furthermore, because the method is based on direct observation, sampling is limited to daylight hours and suitable weather conditions. Resulting distribution maps therefore potentially represent birds of all ages and breeding status and reflect space use during daylight hours and relatively good weather conditions. Tracking provides location information of individuals through time, during both day and night, and under all weather conditions. Data comes from specific individuals usually of known breeding status and provenance. However, the duration over which tracking data can be collected for an individual is limited by deployment constraints (e.g. bird welfare considerations, attachment and retrieval method, battery life), and financial or logistical constraints limit the numbers of individuals which can be tracked in any one year. Therefore, resulting distribution maps typically represent distributions of breeding adults under all light and weather conditions, but often during a concentrated period of the breeding season from a specific sample of colonies and individuals. The latter can lead to concerns regarding representativeness of the resultant sample of tracks and derived maps of space use [19].

Each method therefore comes with its own strengths and weaknesses (depending on the questions being asked) so there is value in the integration of different data sources and several approaches to do so exist. A robust statistical approach is to build hierarchical models applied to transect and tracking data collected systematically to fulfil a common goal, however few studies to date have used this method (but see [20]). An alternative is to build separate models for each dataset and use a model average or the best fitting model to make a single prediction across the study area but we know of no examples where this has been done. The most common approach has been to make a comparison (usually qualitative) of the estimated spatial distributions resulting from separate models for each dataset and use these in a complementary way e.g. [18, 21–26]. This latter approach can offer a pragmatic and relatively simple way to maximise interpretation of different datasets for conservation managers, and is also useful in situations where the original raw data are unavailable or were not designed for more sophisticated statistical integration techniques.

Since 2010, four initiatives have resulted in the generation of distribution maps for seabirds within UK waters during the breeding season. Three of these used transect data (Kober et al. (2010) [27] which was UK-wide; ‘SeaMaST II’, an unpublished updated version of Bradbury et al. (2014) [28] which covered English Waters; and Bradbury et al. (2017) [29] which covered Scottish, Welsh and Northern Irish waters) and one used tracking data (Wakefield et al. (2017) [16], UK-wide). Here, we compare these marine distribution maps for the only four species of breeding seabird which were common across all four studies: common guillemot *Uria aalge* (a pelagic, deep diving auk), hereafter ‘guillemot’; razorbill *Alca torda* (a pelagic, shallow diving auk); black-legged kittiwake (a pelagic, surface feeder), hereafter ‘kittiwake’; and European shag (a coastal, benthic feeder), hereafter ‘shag’.

The main applications for these distribution maps have been to investigate species’ dispersion patterns to better understand their ecological requirements and to identify high density areas suitable for targeting conservation efforts (e.g. identifying protected sites and areas of greater vulnerability) (see Methods). So, as conservation practitioners, we wished to investigate how consistent the maps were in these respects. We recognised that comparisons could be influenced by multiple differences in the properties of the maps due to the sampling process (see above) as well as the modelling process used to derive each map. Given these multiple and potentially confounding differences, our comparison work did not seek to test a series of hypotheses regarding the manner in which the distribution maps would agree or disagree and the possible causes of those (dis-)agreements. Instead, our objectives were to quantitatively assess (i) how well the maps agreed in the location of high use areas, and (ii) the degree of similarity in the mapped distribution patterns.

## Methods

### Data sources and processing

The four maps used in our comparisons were derived from various sources and had already been used for a variety of purposes (Table 1). Kober et al. (2010) [27] used Poisson Kriging to model boat transect data collected between 1980–2004 and generated distribution maps for 26 seabird species across British Fisheries Limits, subsequently used to identify hotspots that might be considered for designation as marine Special Protection Areas (SPAs) [30]. The same boat survey data were supplemented with more recent boat transect data (up to 2014) and aerial transect data (collected between 2000–2013), and modelled for 32 seabird species using Generalised Additive Models for English waters (Bradbury et al. (2014), updated [28]) or Generalised Estimating Equations for the rest of UK waters (Bradbury et al. (2017) [29]). These were modelled as part of two separate assessments of the vulnerability of seabirds to wind farms and by-catch respectively (the latter was combined with the former to generate a single UK-wide map for the by-catch vulnerability assessment). Finally, Wakefield et al. (2017) [16] applied mixed-effects generalised linear models (GLMMs) to tracking data collected for four seabird species breeding at 12–20 UK colonies between 2010–2014 to model habitat use as a function of habitat availability, accessibility and proxies of intra-specific competition. These models were used to produce the first UK-wide at-sea distribution maps for seabirds derived from tracking data, to help highlight areas most warranting conservation measures. Thus, each map varied in several properties relating to survey method, spatial and temporal coverage, analytical technique and output format (Table 1 and S1 Table) but all reflected usage by the species’ population of a given grid cell relative to other grid cells. In all cases, data across years were pooled prior to analysis to produce a single map per species/season, and all of the transect-based maps had adjusted densities for the decline in detectability at increasing distance from the transect line. Hereafter we refer to these four outputs according to the underlying sampling method, namely: ‘boat-only transects’ [27]; ‘boat/aerial transects restricted to English waters’ ([28] updated, though note some Welsh waters are included); ‘boat/aerial transects restricted to Scottish, Welsh and Northern Irish waters’ [29]; and ‘tracking’ [16]. The maps derived from transect data were available for different seasons, while those derived from tracking data were only available for the breeding season. We only compared breeding season maps, noting that there was still variation in the months covered (Table 1).

**Table 1:** The properties of each data source used for the comparisons. S1 Table provides detail on the covariates used in the models.

Property	Data Source			
	Boat-only transects (Kober et al. 2010) [27]	Boat/Aerial transects (‘SeaMaST II’, an unpublished updated version of Bradbury et al. (2014) [28], held by Natural England)	Boat/Aerial transects (Bradbury et al. 2017) [29]	Tracking (Wakefield et al. 2017) [16]
Region of Sea Covered	British Fisheries Limits	English territorial waters	Scottish, Welsh and Northern Irish waters	Various, but includes all (kittiwake) or most (other species) of British Fisheries Limits
Data used	Boat transect data from ESAS database held by JNCC (see [31])	Boat transect data from ESAS database and from Crown Estate Data Catalogue; visual aerial surveys from WWT Consulting database	Boat transect data from ESAS database and from Crown Estate Data Catalogue; visual aerial surveys from WWT Consulting database and JNCC database; digital video aerial surveys (west coast of Lewis, Outer Hebrides)	Tracking data from the ‘FAME’ and ‘STAR’ projects, held by RSPB
Sampled years	1980–2004	Boat: 1979–2014, Aerial: 2001–2013	Boat: 1979–2013, Aerial: 2000–2013	2010–2014
Seasonal time period	May-June (Guillemot/Razorbill); May–Sept (Kittiwake); March-Sept (Shag)	April—Sept	April-Sept (no seasonality for Shag)	May-June
Time of day	Day time only	Day time only	Day time only	Day and night
Weather dependent data collection	Yes	Yes	Yes	No
Model used	Poisson Kriging: estimation based on similarity of data points.	GAM: estimation based on covariate relationship, with a soap film smooth of the coastline	GEE-CRESS SALSA: estimation based on species-specific covariate relationship with a spatially explicit smooth	GLMM (estimation based on species-specific covariate relationship) (i.e. no smooths)
Errors	Standard errors based on survey effort	Model derived CV (doesn’t account for autocorrelation)	Model derived CV (accounts for autocorrelation)	CV derived from parametric resampling (as a way to account for autocorrelation)
Output	Density (individuals per km^2^)	Density (individuals per km^2^)	Density (individuals per km^2^)	Mean proportion of time spent per bird per km^2^
Format	Point data	Grid	Gridded Polygons	Raster
Resolution	6x6km	3x3km	3x3km	2x2km (0.5x0.5km for Shag)
Projection	Ordinance Survey	WGS84	WGS84	Lambert Azimuthal Equal Area

To allow quantitative comparisons between each pair of maps, the outputs from the different datasets were resampled to share a common resolution (1km x 1km for all analyses except the Spearman’s Rank correlation, see later), processed into raster format (if not already) and projected to the same coordinate system (Lambert Azimuthal Equal Area in the ETRS_1989_LAEA coordinate system). The resampling meant that all datasets were downscaled, apart from the shag tracking dataset which was up-scaled. Our metrics of overlap and similarity (see below) were such that the replication of values and increased sample size caused by downscaling would not directly influence their outputs. Therefore downscaling allowed us to extract a high level of spatial information across a common grid size. For each comparison, datasets were first clipped to the commonly shared geographic extent. Most notably, this meant that any comparisons including the updated Bradbury et al. (2014) [28] output were restricted to a shared area within English waters, while those including the Bradbury et al. (2017) [29] output were restricted to a shared area within the rest of UK waters. It is important to note that the boat/aerial transect output restricted to English waters excluded usage values with associated coefficient of variation values exceeding 0.5 as these were considered unreliable [28], which further reduced the area for comparison, in particular for razorbill and shag. All processing and analysis of spatial data was conducted in ArcGIS 10.3.1 and R 3.2.1 (using the Raster package version 2.5–8 [32]).

#### Overlap and agreement in the location of high use areas

Before making comparisons of the locations of high use areas, it is first necessary to define areas of high use. Different thresholds, based on the statistical distribution of the usage values, can be used to delineate areas of high use, so for each map we identified those grid cells in which use exceeded a range of percentile thresholds (50^th^, 75^th^, 90^th^, 95^th^ and 99^th^ percentiles), and then made comparisons between each pair of outputs at each threshold. Pair-wise comparisons were not undertaken where the threshold value in either dataset reached zero i.e. no distinction could be made in one or both rasters as to areas of ‘higher use’. For each pair-wise comparison, we calculated the degree of overlap, defined as the proportion of the locations exceeding the x^th^ percentile which were shared between both data sources, such that a value of one would represent complete overlap in where higher use areas were located. This provided a quantitative measure of the amount of overlap between any two maps in the location of higher use areas, and how this varied depending on the threshold used. For comparison, we calculated this same metric in accordance to what we would expect if cells had been randomly assigned to be above or below the threshold, based on simple probabilities, by dividing the expected number of shared locations ((1-percentile)^2^ * size of study area) by the number of cells in the upper percentile of each output ((1-percentile) * size of study area).

In addition to these pair-wise comparisons we also mapped the degree of agreement in the locations of high use areas between all three outputs (although there were four outputs in total, the maximum number of outputs that could agree in any one area was three as the two boat/aerial transect outputs did not overlap geographically). For this purpose we clipped datasets to the three-way shared area in English and in Scottish/Welsh/Northern Irish waters separately, and then used the 95^th^ percentile threshold for each as a nominal representation of the highest use areas. Each grid cell was then categorised according to the degree of agreement regarding whether it exceeded the 95^th^ percentile threshold, i.e. agreement across all three outputs; agreement across each possible combination of two outputs; no agreement (only one output identified the grid cell as exceeding the 95^th^ percentile threshold); and agreement across all three outputs that the grid cell did not exceed the 95^th^ percentile threshold.

#### Similarity of distribution patterns within core areas of use

To quantify the overall degree of similarity between pairs of estimated spatial distributions, we used Bhattacharyya’s Affinity (BA; [33]), a recommended statistic for providing a general measure of similarity between estimates of utilisation distributions, typically representing individuals or populations [34]. This provides a single measure of overall similarity between each pair-wise comparison and ranges from 0 (no similarity) to 1 (identical utilisation distributions) using the calculation: BA = ∑(√V1 x √V2); where V1 = value in 1^st^ raster and V2 = value in 2^nd^ raster.

The BA statistic was calculated using rasters containing only cells which exceeded the 50th percentile, often used to define the core area of use [19]. This allowed a reasonable size of area over which to calculate the similarity index (because the area of overlap reduces markedly with higher thresholds, see Results), while excluding areas of very low usage which are of less interest and may otherwise have complicated the interpretation of the calculation; BA is a useful tool to compare areas of usage, rather than areas of non-usage. Exceptions to using the 50^th^ percentile had to be made for selected pair-wise comparisons for razorbill and shag where the threshold value had already reached zero. In these cases, the ‘core area of use’ was represented by the next highest percentile that ensured the threshold value exceeded zero (this was either the 75^th^ or 90^th^ percentile). To make values appropriate as inputs to the BA formula, which requires the values to sum to one, the data in ‘core use’ rasters were re-scaled (i.e. each grid cell value was divided by the sum of values in the grid).

As the BA statistic provides only a single value for each comparison, we assessed how similarity varied spatially by calculating the proportional contribution of each grid cell to the overall BA value for the area of overlap, where higher values indicated co-occurrence of high usage and lower values indicated either less similarity or co-occurrence of low usage. We used Spearman’s rank correlations to assess how the cell-by-cell proportional contribution to overall similarity in each pairwise comparison varied with distance to the nearest conspecific breeding colony (using minimum distance-by-sea, calculated as per Wakefield et al. (2017) [16]). Before doing this all inputs were resampled to share a common resolution defined by the raster in each pair which originally had the largest cell size, to avoid falsely inflating sample size for this statistical test. Non-parametric tests were used since the data were under-dispersed. We only considered the relative size and direction of the Spearman’s rank test values, not P-values, due to spatial autocorrelation. Spearman’s rank tests were conducted using cor.test in R. Regression lines were plotted using the geom._smooth() function in the R package ggplot2 (version 2.2.1) [35], specified using binomial GLM with an x~y relationship.

## Results

### Overlap and agreement in the location of high use areas

**Fig 1:**
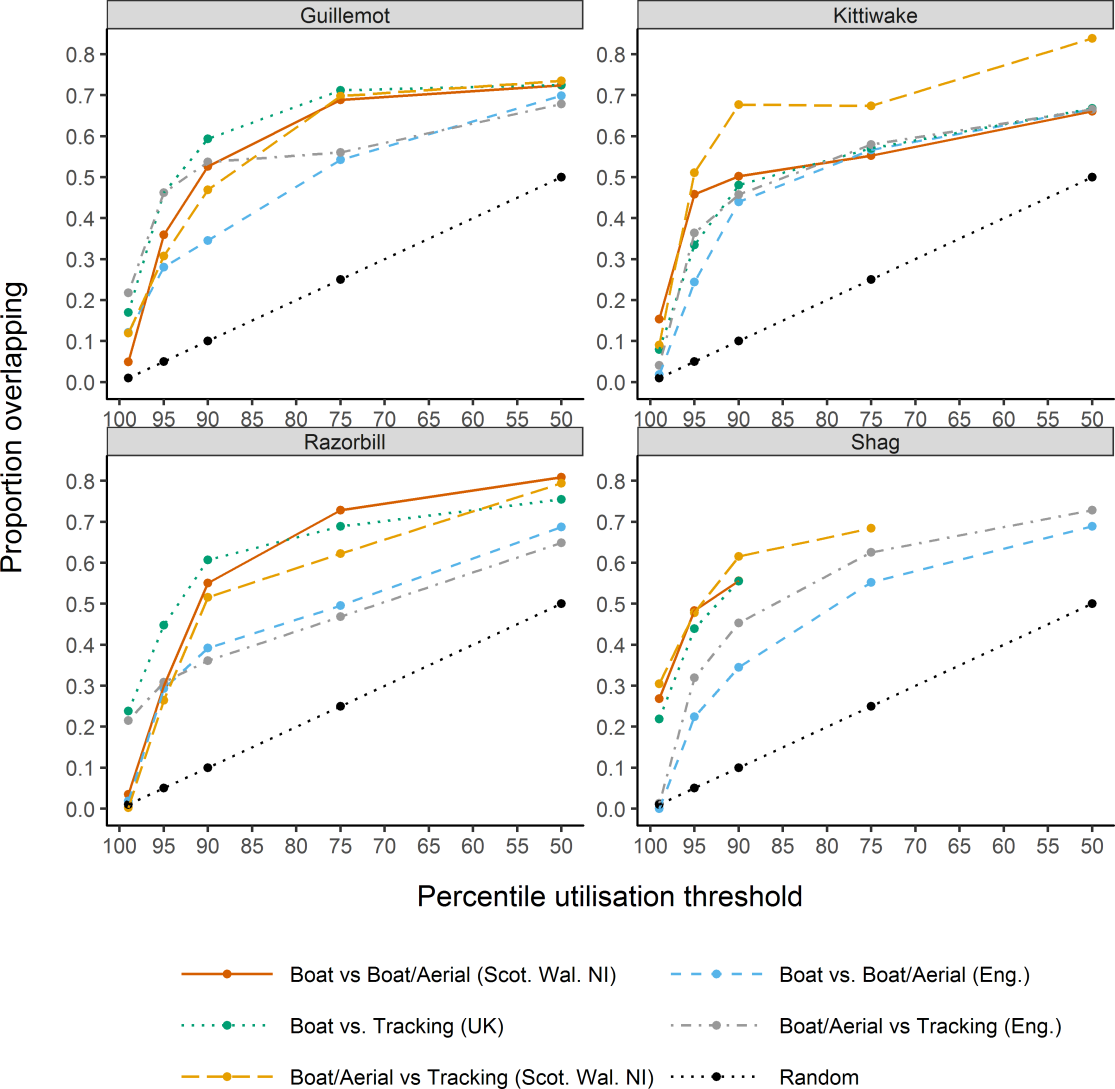
Overlap in high use areas for pair-wise comparisons as the percentile threshold defining ‘high use’ decreases. The black dotted line shows the proportional overlap expected by chance. A value of zero indicates no overlap and a value of one indicates complete overlap.

We made a total of 100 pair-wise comparisons (five for each of the four species, for each of the five thresholds), to assess how overlap varies depending on the threshold used to define high use (Fig 1). In all cases but two, the degree of overlap (the proportion of the locations exceeding the x^th^ percentile which was shared between both data sources) increased as the threshold percentile defining high usage was reduced, with similar patterns of change across all species. Specifically, in every comparison, overlap was lowest when only cells exceeding the 99^th^ percentile were considered (mean proportional overlap across all species and comparisons = 0.13). The degree of overlap increased markedly as the threshold for defining high use was reduced to the 95^th^ or 90^th^ percentile (mean proportional overlap across all species and comparisons = 0.50 at the 90^th^ percentile), but the rate of increase in overlap slowed once thresholds below 90^th^ percentile were considered. Crucially, the degree of overlap was consistently greater than would be expected by chance, except in some cases for the 99^th^ percentile threshold.

Given our interest in areas of highest use and the relatively poor levels of overlap when using the 99^th^ percentile threshold (Fig 1), we present a further investigation of patterns in the degree of overlap in the location of high use areas only for the 95^th^ percentile threshold (28 pair-wise comparisons, seven for each of the four species, see Table 2). The degree of overlap varied among comparisons, with the proportional overlap of high use areas ranging from 0.22 to 0.51 (Table 2). However, there was little difference in the level of overlap within grouped comparisons for: species (mean proportional overlap = 0.38 (guillemot), 0.34 (razorbill), 0.35 (kittiwake) and 0.4 (shag)); regions (mean proportional overlap = 0.32 (English), rest of UK waters (0.39), UK as a whole (0.42)); or data type (mean proportional overlap = 0.38 (tracking / transect comparisons), 0.33 (transect/transect comparisons)).

**Table 2:** Extent of overlap of ‘high use’ areas (cells >95^th^ percentile) in pair-wise comparisons of datasets.

Species	Region	Dataset 1	Dataset 2	‘High use’ area (cells >95^th^ percentile) sampled in each dataset (km^2^)	Area of overlap between datasets in the location of ‘high use’ cells (km^2^)	Extent of overlap of ‘high use’ areas as proportion of area exceeding the 95^th^ percentile
Guillemot	English waters	Boat only	Boat/Aerial	8943	2509	0.28
		Boat only	Tracking	10693	4202	0.39
		Boat/Aerial	Tracking	7794	3601	0.46
	Scottish, Welsh, N. Irish waters	Boat only	Boat/Aerial	21864	7852	0.36
		Boat only	Tracking	20955	8666	0.41
		Boat/Aerial	Tracking	23146	7111	0.31
	UK waters	Boat only	Tracking	31666	14623	0.46
Razorbill	English waters	Boat only	Boat/Aerial	5950	1745	0.29
		Boat only	Tracking	9732	3018	0.31
		Boat/Aerial	Tracking	4652	1435	0.31
	Scottish, Welsh, N.Irish waters	Boat only	Boat/Aerial	23454	7047	0.30
		Boat only	Tracking	21526	9108	0.42
		Boat/Aerial	Tracking	22679	6005	0.26
	UK waters	Boat only	Tracking	31356	14032	0.45
Kittiwake	English waters	Boat only	Boat/Aerial	11741	2866	0.24
		Boat only	Tracking	11868	2851	0.24
		Boat/Aerial	Tracking	11812	4298	0.36
	Scottish, Welsh, N.Irish waters	Boat only	Boat/Aerial	24176	11075	0.46
		Boat only	Tracking	21902	7098	0.32
		Boat/Aerial	Tracking	22632	11552	0.51
	UK waters	Boat only	Tracking	34044	11376	0.33
Shag	English waters	Boat only	Boat/Aerial	419	94	0.22
		Boat only	Tracking	6354	2845	0.45
		Boat/Aerial	Tracking	382	122	0.32
	Scottish, Welsh, N.Irish waters	Boat only	Boat/Aerial	25479	12321	0.48
		Boat only	Tracking	17292	7085	0.41
		Boat/Aerial	Tracking	17674	8453	0.48
	UK waters	Boat only	Tracking	23535	10334	0.44

**Fig 2:**
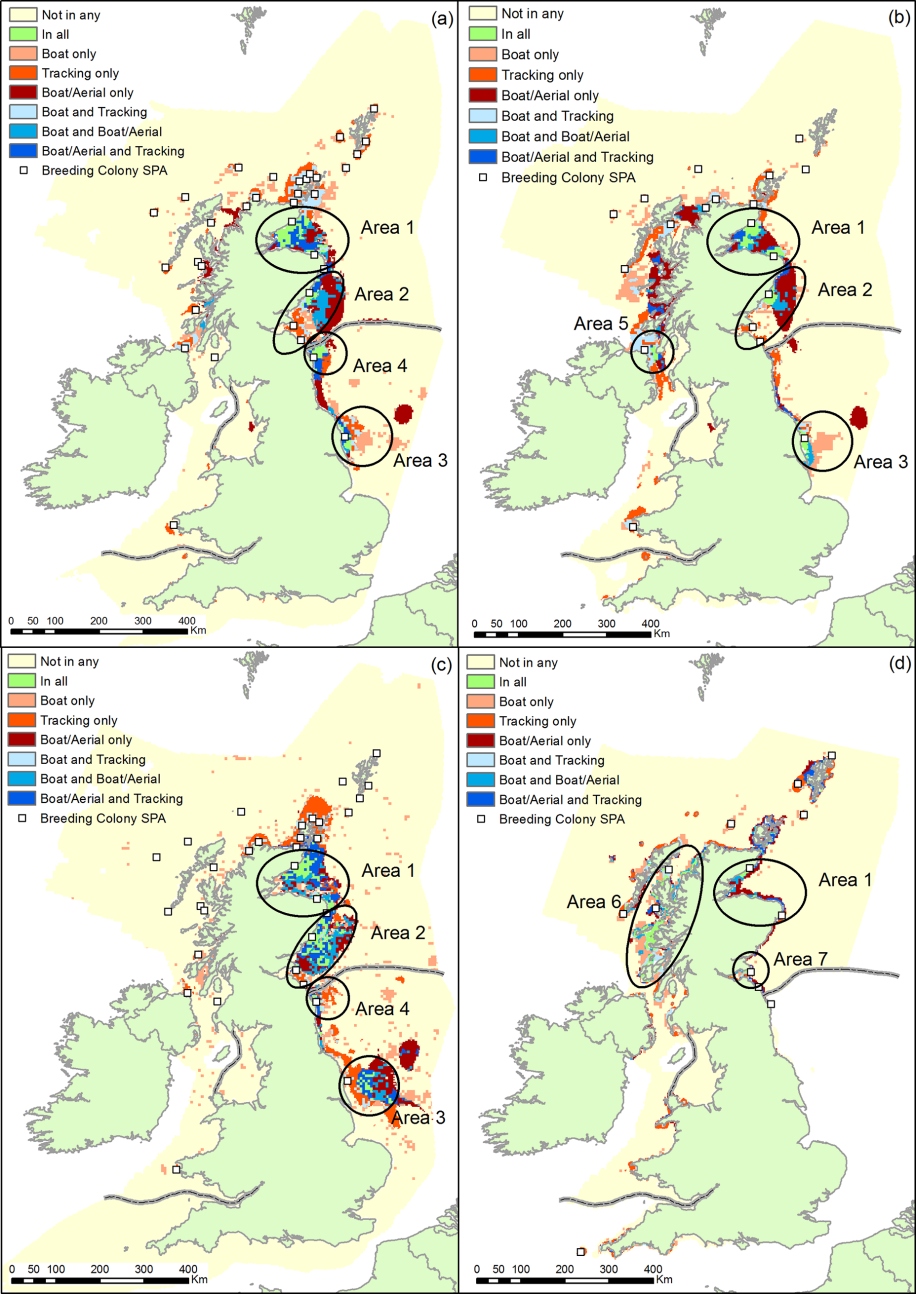
Agreement in locations of ‘high use’ areas (cells > 95^th^ percentile) in different distribution maps. (a) guillemot, (b) razorbill, (c) kittiwake and (d) shag. Colour shading shows degree of agreement: green—all three maps agreed the area was ‘high use’; shades of blue—two maps agreed the area was ‘high use’; shades of red—only one map identified the area as ‘high use’, yellow—all maps agreed the area was not ‘high use’. Grey lines indicate the different regions within which comparisons were made (‘English waters’ and ‘Scottish, Welsh and Northern Irish waters’). Breeding colony SPA locations for which the relevant species is a designated feature are shown (provided by Joint Nature Conservation Committee under Open Government License). European coastline base map provided by the European Environment Agency under ODC-by license.

There were some general similarities among species in terms of broad regions of higher usage (>95^th^ percentile) identified by all three datasets (Fig 2). For guillemot, razorbill and kittiwake, these were: within the Moray Firth (Area 1 in Fig 2A–2C); off the Grampian and Angus coast in east Scotland (Area 2 in Fig 2A–2C); and an area off the Yorkshire coast adjacent to Flamborough Head and the Filey Coast SPA (Area 3 in Fig 2A–2C). However, the latter area of agreement for guillemot and razorbill was coastal while for kittiwake, it was further offshore around the Dogger Bank area. For guillemot and kittiwake there was also agreement among all three datasets that there was a high use area off the Northumberland coast adjacent to the Farne Islands SPA (Area 4 in Fig 2A and 2C) held high densities, though this was more extensive for guillemot than for kittiwake. For razorbill, there was an additional high use area agreed across all three datasets at the narrowest part of the North Channel, between north-east Ireland and south-west Scotland, (Area 5 in Fig 2B). For shags, high use areas were much more restricted to the coastline than for the other species. The main region where there was shared agreement among all three datasets for this species was in scattered areas along the west coast of the Scottish mainland (Area 6 in Fig 2D). Other, much less extensive areas of agreement for shag included the Moray Firth (e.g. off the North Caithness Cliffs SPA) (Area 1 in Fig 2D) and along the Fife coast opposite the Isle of May, part of the Forth Islands SPA (Area 7 in Fig 2D).

### Similarity of spatial distribution patterns within core areas of use

**Fig 3:**
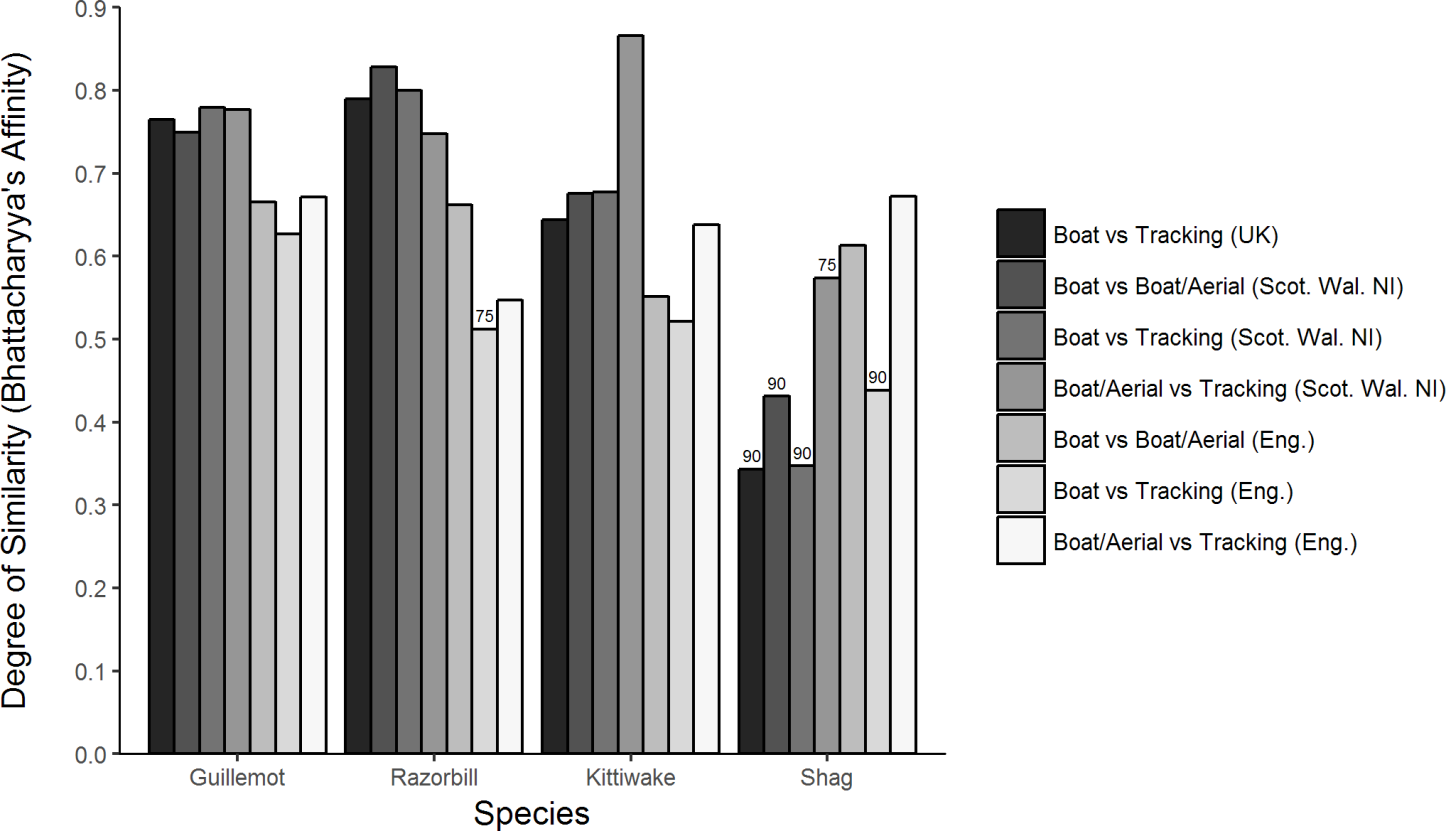
The degree of similarity between pair-wise comparisons of spatial distribution patterns. Similarity was measured by Bhattacharyya’s Affinity which ranges in value from 0 (no similarity) to 1 (identical utilisation distributions). These were calculated for cells > 50^th^ percentile, except for selected pair-wise comparisons for razorbill and shag (see labelled bars), which were calculated for cells > 75^th^ or 90^th^ percentile (see methods).

The overall similarity among datasets in the pattern of use across core areas (grid cells exceeding the 50^th^ percentile) was typically high (BA>0.5) for all species, with the exception of shag where BA values for four of the seven pair-wise comparisons were <0.5 (Fig 3). Note however that all four of these were comparisons using data with a 90^th^, rather than 50^th^, percentile threshold so similarity is expected to be lower. The BA value reached a maximum of 0.87 for the comparison for kittiwakes between boat/aerial transects and tracking datasets restricted to Scottish, Welsh and Northern Irish waters. However, BA values varied little within grouped comparisons for regions (mean BA = 0.59 (English waters), 0.69 (rest of UK waters), 0.64 (UK as a whole) or data type (mean BA = 0.64 (tracking / transect comparisons), 0.65 (transect/transect comparisons)).

**Fig 4:**
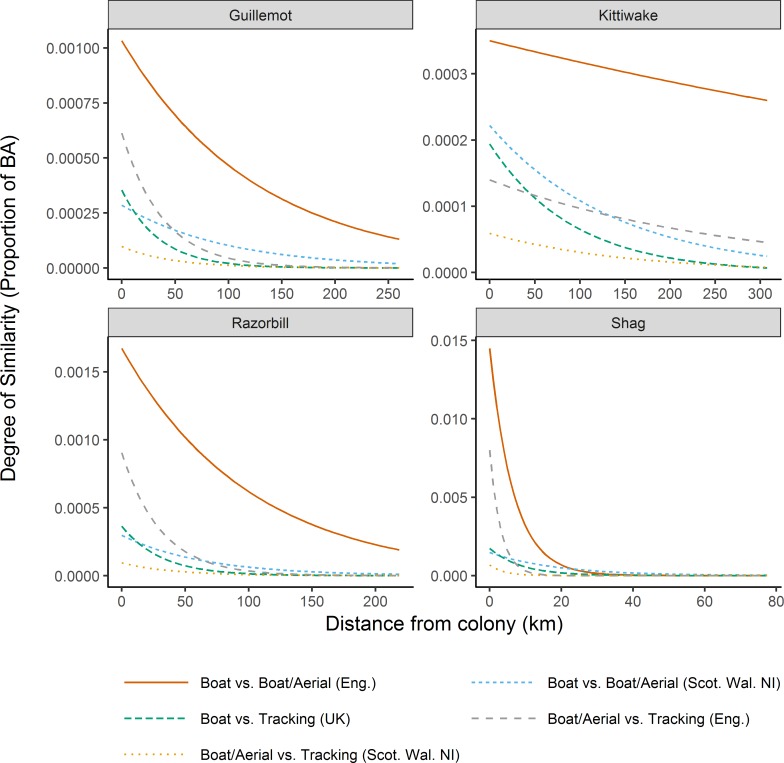
The degree of similarity between datasets in relation to distance from the nearest conspecific colony. Similarity was measured as the proportional contribution to the overall Bhattacharyya’s Affinity (BA) value. BA scores compare areas of usage rather than non-usage, so while higher values indicate co-occurrence of high usage, lower values can reflect low usage in one or both datasets.

We found that for almost all pair-wise comparisons, similarity was greatest closest to the nearest conspecific colony and declined with distance from it, with the rate of decline greatest closer to the colony (Table 3, Fig 4). The only two exceptions to this were the pair-wise comparisons that included the boat/aerial transect datasets for kittiwake within English waters; more generally, the strength of the relationship between similarity and distance from the colony was least pronounced in kittiwake.

**Table 3:** Spearman’s rank correlations between the contribution of each grid cell to the overall BA similarity value for each pair-wise comparison and the distance of that cell from the nearest colony.

Region	Comparison	Guillemot	Razorbill	Kittiwake	Shag
England	Boat only vs boat/aerial	-0.597	-0.647	-0.060	-0.635
	Tracking vs boat/aerial	-0.564	-0.643	0.057	-0.653
Scotland and Wales	Boat only vs boat/aerial	-0.381	-0.407	-0.242	-0.386^1^
	Tracking vs boat/aerial	-0.518	-0.481	-0.200	-0.741^2^
UK	Boat only vs Tracking	-0.554	-0.581	-0.329	-0.548^1^

BA statistics were calculated for core areas (grid cells > 50^th^ percentile), except where highlighted (^1^ = grid cells exceeding the 90^th^ percentile; ^2^ = grid cells exceeding the 75^th^ percentile; see methods).

## Discussion

To our knowledge we have undertaken the first quantitative comparison of population-level seabird marine distribution maps derived from tracking and transect samples at a UK scale. This has allowed us to quantify overlap and agreement in the location of high use areas and the degree of similarity in the mapped distribution patterns. The majority of high use areas identified across all data sources were within inshore waters (<12nm from shore). While shag showed the most coastally restricted of these, some agreed high use areas for kittiwake extended into offshore waters, reflecting differences in their foraging ranges [36]. Four broad regions were consistently identified as high use areas across multiple species: the Moray Firth, and areas off the Grampian / Angus, Northumberland and Yorkshire coasts. In general, the locations of these areas make biological sense as they tend to be close to known breeding colonies, or in the vicinity of shallow sand banks (e.g. Dogger Bank, Wee Bankie, Marr Bank, Smith Bank) which are known to be important sandeel spawning areas [37, 38]. These areas are of particular conservation interest as they are subject to pressure from various human activities including an industrial sandeel fishery (Dogger Bank [39]), several operational or planned renewable energy developments [40], and have all been identified as being at relatively high risk to seabird bycatch from various types of fishing gear [29]. While our analysis was not designed to identify marine protected areas, some of the high use areas consistently identified across the datasets (parts of the Moray Firth, the Firth of Forth, and off the Northumberland coast) are being considered as marine Special Protection Areas, or have recently been designated as such [41–44].

Almost all pair-wise comparisons reveal a far greater degree of overlap in used locations at all percentile thresholds examined than would be expected by chance. This indicates that all the datasets captured a common signal in the spatial dispersion of the four species. Proportional overlap in the location of high use areas increased as the percentile threshold defining high use was lowered indicating that the signal becomes stronger when progressing the search for overlap from locations of highest use towards those of core use. At the 99^th^ percentile threshold the level of overlap reached a maximum of just 30% and in some cases overlap was no better than random. In contrast, for the 95^th^ percentile threshold, overlap between high use areas was between 22% and 51%, compared to an expected degree of overlap of just 5%. Our analysis indicated that increasing the threshold of ‘high use’ above the 95^th^ percentile severely risks the reliability of any positive corroboration results, which could potentially be no better than random and risks failure to corroborate truly important areas due to application of too stringent a definition of ‘high use’. The definition of ‘high use’ therefore requires very careful consideration particularly in the context of applying an integrated approach to site identification that relies on corroboration of ‘high use’ across independent datasets.

Within the broader sea areas defined by the 50^th^ percentile i.e. ‘core’ areas, measures of similarity in spatial use by all species were typically high (BA > 0.5). Within these ‘core’ areas distance to the nearest conspecific colony appeared to be an important influence on how well the outputs compared.

For all species, the proportional contribution to overall similarity was greatest closest to the nearest conspecific colony and declined with distance from the nearest conspecific colony (or there was increasing similarity in respect of there being relatively low use further from colonies; low BA values indicate either scenario). This most likely reflects the fact that seabird ecology during the breeding season is heavily influenced by central place foraging constraints. The only exceptions to the general pattern were two pair-wise comparisons for kittiwakes in English waters where there was clear evidence of high use in large areas relatively far offshore from the nearest colony on the Yorkshire coast (Fig 2C).

Our comparisons focussed on overlap, agreement and similarity, rather than differences, between maps due to the difficulties associated with disentangling multiple ecological and statistical properties that might lead to differences. However, one of the most obvious differences in the biological information contained within the tracking dataset as opposed to the various transect datasets is that the former captures information on the spatial distribution of breeding individuals only. The locations of high use areas from outputs based on samples that potentially included non-breeding birds (i.e. transect datasets, particularly those which included surveys carried out later in the season during post-breeding dispersal offshore) might therefore be expected to be less concentrated around breeding colonies than the tracking datasets. However, although transect samples did identify high use areas further offshore that were not corroborated by tracking data (e.g. dark red/brown areas in Fig 2A–2C), there was no quantitative evidence that tracking- and transect-derived maps showed less overlap (in the location of high use areas), or were less similar to each other (in their core distribution patterns) than was the case for any two transect-derived maps, despite the more fundamental differences between the former data types. There are several potential reasons for this finding. Firstly, non-breeders may not have a great influence on the at-sea distributions derived from transect samples, either because they are to some extent also subject to central place foraging (e.g. they are prospecting breeding sites, e.g. [45] and their distributions, therefore, resemble those of breeding birds), or because they are so dispersed that transect-derived maps of distribution during the breeding season are overwhelmingly dominated by aggregations of breeding birds. In addition, non-breeders are thought to generally comprise a smaller proportion of the overall population than breeders [46, 47] (though notably one study estimates that the ratio of immature birds to breeding adults is 1.31 for shag [47]). Secondly, the differing sample periods of the datasets may play a role if distributions have changed over time. For example if breeding birds range more widely during times of food stress and, as indicated in [48], there has been increasing food stress in recent years, then the more recently sampled transect and tracking at-sea distributions may show dispersion patterns more closely resembling each other than either resemble those derived from less recent transect sampling. Thirdly, choice of modelling technique has been shown to influence the estimated spatial distribution and therefore which areas are identified as important [49]. While the boat-only transects were modelled based only on the inherent spatial autocorrelation in the data, models for the other three outputs all included covariates. None of these three possibilities are mutually-exclusive nor possible to discount or demonstrate easily.

Conservation efforts, in particular the designation of protected sites and assessment of the impacts to anthropogenic activities, rightly face high levels of scrutiny and it is important that the identification of important or vulnerable areas for marine birds is supported by as strong an empirical evidence base as possible. Corroboration by more than one independent data source is one way to maximise confidence that protective measures are put in the right place [21, 50]. Although the datasets we compared cannot be considered truly independent, locations where we have found total agreement that they are highly used regardless of the survey platform, age of data, population sampled and modelling applied, are areas in which there can be high confidence of their persistent importance during the breeding season for the species concerned. Areas such as these where there is shared agreement in their importance across differing data sources merit prioritisation for consideration of conservation measures and particular scrutiny when assessing potential impacts of any current or planned anthropogenic activities. Notwithstanding this, when interpreting the results from the comparisons, it is important to include consideration of the spatial distribution of uncertainty associated with each modelled output. There will be lower confidence that agreed areas of high use are truly important if there is high uncertainty in one or both of the underlying datasets compared to those where uncertainty is low in both. However, although error information is available for all of the datasets, the derivation and meaning of the metrics varied among them (see Table 1) so the relative magnitude of the errors may not be equivalent between data sets. Therefore, we were unable to test how levels of error affected the degree of agreement in our pair-wise comparisons.

This study has highlighted the value of considering multiple data sources to inform marine conservation and management decisions and developed a protocol to do so using quantitative comparisons that could be extended further to a wider range of species, locations and geographic scales. However, much progress could be made in the future by controlling for some of the differences that have hampered our ability to interpret our results. A priority would be to undertake concurrent sampling by both transects and tracking within the same area (controlling for geographic and temporal differences) with sampling and analysis designed specifically with the aim of using differences between the resulting outputs to distinguish breeder and non-breeder distributions. This would improve our understanding of potential asymmetries in how pressures might impact different components of the population and where protective measures for each are best targeted.

## Supporting information

S1 Table Covariates used in the models underlying each of the mapped outputs used in the comparisons. (DOCX)
