# Supplementary material for: Comparing marine distribution maps for seabirds during the breeding season derived from different survey and analysis methods
Source: PLoS One. 2018 Aug 29;13(8):e0201797. doi: 10.1371/journal.pone.0201797 (PMC6114294; doi:10.1371/journal.pone.0201797)
Supplement: S1 Table — (DOCX) [file pone.0201797.s001.docx]

**S1 Table. Covariates used in the models underlying each of the mapped outputs used in the comparisons.**

|  | **Boat-only transects**  **(**[**27**](#_ENREF_27)**)** | **Boat/Aerial transects**  **(‘SeaMaST II’, an unpublished updated version of (**[**28**](#_ENREF_28)**), held by Natural England)** | **Boat/Aerial transects**  **(**[**29**](#_ENREF_29)**)** | | | **Tracking**  **(**[**16**](#_ENREF_16)**)** | | | |
| --- | --- | --- | --- | --- | --- | --- | --- | --- | --- |
| **Covariate** | All four species | All four species | Guillemot/ Kittiwake | Razorbill | Shag | Guillemot | Razorbill | Kittiwake | Shag |
| **Poisson kriging using spatial information in the data** | x |  |  |  |  |  |  |  |  |
| **Latitude/Longitude** |  | x | x | x | x |  |  |  |  |
| **Distance to coast** |  | x |  |  | x | x |  |  |  |
| **Depth** |  |  | x |  |  |  |  |  |  |
| **Season** |  |  | x | x |  |  |  |  |  |
| **Distance to colony** |  |  |  |  |  | x | x | x | X |
| **Cumulative area at distance from colony** |  |  |  |  |  | x | x | x | x |
| **Intra-specific competition** |  |  |  |  |  | x | x | x | x |
| **Gravel** |  |  |  |  |  | x |  |  | x |
| **Sand:mud** |  |  |  |  |  |  | x |  |  |
| **Thermal front gradient density** |  |  |  |  |  | x |  |  |  |
| **Sea surface temperature** |  |  |  |  |  |  | x | x |  |
| **Seabed slope** |  |  |  |  |  |  | x | x |  |
| **Stratification** |  |  |  |  |  |  |  |  |  |
| **Mean potential energy anomaly** |  |  |  |  |  |  |  |  | x |
